# Comparison of the effects of different blood conservation techniques in elderly patients undergoing total hip arthroplasty

**DOI:** 10.4314/ahs.v23i3.59

**Published:** 2023-09

**Authors:** Yuanxing Cai, Xin He, Qinghao Cheng

**Affiliations:** Department of Anesthesiology, Emergency General Hospital, Beijing, China

**Keywords:** Hip arthroplasty, blood transfusion, autologous, hemodilution, transfusion of red blood cells, platelet activating factors

## Abstract

**Background:**

To probe into the influences of different blood conservation techniques on the postoperative coagulation function and prognosis of elderly patients receiving Total Hip Arthroplasty (THA).

**Methodology:**

A total of 60 patients were randomly divided into Autologous Blood Transfusion (ABT) group (n=30) and ANH group (n=30). For patients in the ABT group, an autologous blood recovery machine was used to recover, wash and filter the surgical field blood. For those in the Acute Isovolumic Hemodilution (ANH) group, blood was collected preoperatively from the central vein and stored in a citrate anticoagulant blood storage bag, while the same amount of hydroxyethyl starch was injected into the peripheral vein to dilute the blood. After Mai bleeding steps of the operation were completed, the autologous blood of patients was transfused back in both groups. The clinical indicators of patients in each group were observed.

**Results:**

48 h after operation, the ANH group obtained a higher level of hemoglobin (Hb), shorter Activated Partial Thromboplastin Time (APTT), and a lower expression rate of platelet activating factor CD62P than the ABT group.

**Conclusion:**

The ANH group exhibits higher content of hemoglobin and fewer platelet (Plt)activating factors produced than the ABT group, while no significant difference in the shortened length of hospital stays is found.

## Introduction

With the social and economic development of China, population aging has become increasingly serious. The number of elderly patients that need total hip arthroplasty (THA) due to hip fractures experiences an upward trend annually. One of the common complications after THA is perioperative blood loss, which may be attributed to multiple factors.

1. Currently, autologous blood transfusion (ABT) and acute isovolumic hemodilution (ANH) are widely used techniques for blood conservation in clinical practice.

2. The specific steps of these methods are collecting the blood of patients during or before the operation, washing or storing it appropriately, and transfusing it back to the body of patients after surgical bleeding procedures are basically completed, thus achieving the goals of supplementing red blood cells and improving the oxygen carrying capacity. The collection of autologous blood can not only avoid various risks of transfusing allogeneic blood [e.g., blood-borne infectious diseases like hepatitis and human immunodeficiency virus (HIV) infection, as well as immune reactions], but also solve the problem of insufficient banked blood, thereby getting rapid development in clinical applications.

In this research, the influences of ABT and ANH on prognosis of elderly patients with approximate bleeding volumes during THA were explored by observing the changes of such indexes as intraoperative and postoperative vital signs, coagulation function, platelet (Plt) activating factors (PAC-1 and CD62P) and lactate (Lac), as well as the length of hospital stays of patients when different blood conservation techniques were used, so as to perfect the blood conservation programs for elderly patients undergoing THA.

## Patients and methods

### Patients

Patients who intended to receive THA in the Department of Orthopedics in our hospital from January 2018 to December 2019 were enrolled.

**The inclusion criteria were set as follows:**

1. Patients aged 60-85 years old, ASA I-III grade,
2. Those having estimated intraoperative blood loss ≥600 mL or 12% of the systemic blood volume,
3. Those with preoperative hemoglobin (Hb) ≥125 g/dL, hematokrit (Hct) ≥35%, and normal platelet counts and coagulation function, and
4. Those voluntarily signing the informed consent for this experiment before the operation.

**The exclusion criteria involved:**

1. Patients suffering from liver, kidney or immune system diseases before the operation,
2. Those suffering from viral and bacterial infections before the operation,
3. Incomplete retention of clinical information on patients,
4. Those having a history of thrombosis, cerebral infarction, or heart failure, or suffering from paraplegia, or
5. Those having a history of related drug allergies.

A total of 60 patients (37 males and 23 females) whose age, general condition and medical history met the inclusion criteria aforesaid were enrolled and evenly divided into ABT group and ANH group using a random number table. The age and gender ratio showed no significant differences between the two groups (p>0.05). The approval from the Ethics Committee of Emergency General Hospital and the informed consent signed by all enrolled patients were obtained.

## Methods

Patients fasted for 6hours before the operation. After admission into the operation room, routine monitoring of a 5-lead electrocardiogram (ECG), non-invasive blood pressure (NIBP), systemic pulse oxygen saturation (SPO_2_), and end-tidal carbon dioxide concentration (ETCO_2_), and anesthesia depth monitoring [bispectral index (BIS)] were conducted. Then, intravenous access was established to supplement the fluid lost during preoperative fasting. Next, radial artery catheterization was conducted before induction for invasive arterial blood pressure (ABP) monitoring, and regular detection of blood gas during the operation was carried out. Furthermore, 0.3 mg of scopolamine or 0.5 mg of atropine was injected intravenously, and pure oxygen was inhaled for 5 min before induction. Afterwards, intravenous induction was performed according to the assessment results of the cardiopulmonary function of patients.

Midazolam (0.06 mg/kg), sufentanil (0.4 *u*g/kg), etomidate (0.2 mg/kg), and cisatracurium besylate (0.2-0.3 mg/kg) were successively used for induction. After induction, a double-tube non-inflatable laryngeal mask was placed via the mouth and connected to a ventilator, which controlled ventilation by the volume to maintain ETCO_2_ at 35-45 mmHg. After the induction of general anesthesia, the right subclavian or internal jugular vein catheterization was carried out, combined with the monitoring of central venous pressure (CVP). Moreover, the injection of propofol (4-6 mg·kg^-1^·h^-1^) and remifentanil (0.2-0.3 *µ*g·kg^-1^·min^-1^ and intermittent bolus injection of cisatracurium besylate were performed to maintain the anesthesia depth at the BIS of 40-60.

In ABT group, an autologous blood recovery machine (BW-8200B, Wandong Health Resources Inc.) was used to recover the autologous blood of patients during the operation. The heparin saline (200 mg of heparin per 500 mL of normal saline) was used to flush the recovery system before blood recovery. After the surgical incision, the recovery of the surgical field blood started, during which the dripping rate of heparin saline was adjusted to maintain the ratio of dripping amount of heparin to the blood drawing volume at 1:5. Centrifugation, washing and recovery started automatically when the total recovery volume in the blood storage tank reached 800 mL. If this amount was not met after the main bleeding steps of the operation were completed, centrifugation and washing were performed manually. Afterward, the washed red blood cells were all extracted into the finished bag and transfused back by passing through a leukocyte filter. The rate of transfusion was adjusted based on the vital signs, bleeding volume and bleeding rate of patients.

In ANH group, blood collection from the central vein was performed after induction of general anesthesia. Next, estimated blood volume (mL) was calculated based on the following formula: estimated blood volume (mL)= (preoperative Hct - intended dilution target Hct) / (preoperative Hct + intended dilution target Hct) ×2× estimated total blood volume (70 mL/kg for males and 65 mL/kg for females). The blood collection speed set should ensure the fluctuation range of hemodynamic indexes <20%. During blood collection, the same amount of hydroxyethyl starch was injected through the subclavian vein or the internal jugular vein for hemodilution. For all patients in the ANH group, the target Hct ≥ 30% was controlled.

### Observation of indexes

The changes in vital signs, intraoperative bleeding volume and transfusion volume, preoperative and postoperative hemoglobin, platelet, platelet activating factors (PAC-1 and CD62P), prothrombin time (PT), activated partial thromboplastin time (APTT), fibrinogen (Fib), D-dimer, Lac, operation time, blood transfusion reaction and length of hospital stays were observed.

The platelet activating factors were determined as follows: The venous blood (4-6 mL) was collected from patients in ABT group immediately before induction of anesthesia (T_0_), before transfusion of washed autologous red blood cells (T_1_), at 1 h after transfusion of washed autologous red blood cells (T_2_), at 24 h after the operation (T_3_), and at 48 h after the operation (T_4_), respectively, as well as patients in ANH group immediately before induction of anesthesia (T_0_), at 30 min after ANH (T_1_), at 1 h after ABT (T_2_), at 24 h after the operation (T_3_), and at 48 h after the operation (T_4_), respectively. Then, venous blood collected was evenly placed into an anticoagulation tube containing ethylene diamine tetraacetic acid (EDTA) and that containing sodium citrate 1:9, respectively. Moreover, the expression levels of PAC-1 and CD62P were detected by three-color fluorescence flow cytometry (FCM), and data collection and analysis were carried out with Cell Quest software. The content of PAC-1 and CD62P was expressed as the percentage of positive platelets.

### Statistical analysis

SPSS 21.0 software was applied to statistical analysis, the obtained measurement data were expressed as mean ± standard deviation (^-^χ±s), and two independent samples t-test was used for intergroup comparisons. Moreover, χ^2^ test was performed on the obtained count data. Statistical significance was defined as P<0.05.

## Results

### Hemodynamic parameters Patients in both groups underwent total hip arthroplasty under general anesthesia

There was no significant difference in operation time and intraoperative blood loss between the two groups (*P* > 0.05). Blood transfusion volume was recorded in ABT group and ANH group. Statistical calculation There was no significant difference between ABT group and ANH group (*P* > 0.05) as shown in Table 2. Hemodynamic parameters, including mean arterial pressure (MAP) and heart rate (HR), were not significantly different between groups before induction, before and after transfusion, 24 hours after transfusion and 48 hours after surgery (*P* > 0.05) as shown in Table 1.

**Table 1:** Comparison of vital signs, blood loss, blood transfusion volume and operation time at each time point between the two groups

Group		ABT Group	ANH Group	t	*P*
MAP (mmHg)	T0	99.9 ± 13.3	101.8 ± 12.4	-0.584	0.561
	T1	78.3 ± 13.3	81.2 ± 12.9	-0.844	0.402
	T2	79.7 ± 14.3	76.5 ± 12.7	0.931	0.356
	T3	80.8 ± 13.3	80.9 ± 13.2	-0.046	0.964
	T4	79.0 ± 13.7	77.5 ± 15.7	0.380	0.705
	T0	76.0 ± 8.8	73.1 ± 9.0	1.261	0.212
HR, bpm	T1	84.9 ± 8.4	82.8 ± 9.5	0.937	0.353
	T2	86.6 ± 10.4	86.8 ± 8.6	-0.054	0.957
	T3	84.4 ± 9.3	84.1 ± 8.7	0.129	0.898
	T4	82.3 ± 8.8	83.4 ± 8.3	-0.481	0.632
Bleeding volume (ml)		693.1 ± 106.7	718.3 ± 115.8	-0.876	0.385
Blood transfusion volume (ml)		313.6 ± 34.8	297.0 ± 47.9	1.540	0.129
Operative Time (min)		78.2 ± 11.1	77.0 ± 10.2	0.436	0.664

**Table 2:** Comparison of hemoglobin and coagulation function between the two groups

Group	ABT Group	ANH Group	t	*P*
Pre-operative Hb (g/L)	136.1 ± 19.2	139.7 ± 10.7	-0.907	0.368
48h H b (g/L)	102.7 ± 15.5	117.2 ± 13.4	3.868	<0.001 *
Pre-operative PT (s)	13.5 ± 1.1	13.3 ± 1.2	0.631	0.530
48 h PT (s)	13.8 ± 2.0	14.3 ± 1.2	-1.212	0.230
Pre-Operative APTT (s)	37.0 ± 8.0	36.4 ± 4.9	0.389	0.699
Post-operative 48 h APTT (s)	36.8 ± 4.2	31.9 ± 6.7	3.378	0.001^Δ^
Pre-operative F ib (g/L)	355.3 ± 4 8.4	322.5 ± 38.4	1.559	0.124
Post-operative F ib (g/L)	303.8 ± 31.6	317.9 ± 59.9	-1.140	0.259
Pre-operative D- dimer (ug/ml)	0.25 ± 0.03	0.29 ± 0.12	-1.601	0.115
48 h D- dimer (ug/ml)	0.39 ± 0.03	0.37 ± 0.07	0.812	0.420

* Note: At 48 h after operation, hemoglobin in ANH group was higher than that in ABT group

*P* < 0.05; Δ at 48 h after operation, APTT in ANH group was lower than that in ABT group, *P* < 0.05

## Laboratory indicators

### Hemoglobin and coagulation

There was no significant difference in preoperative hemoglobin between the two groups (*P* > 0.05), and postoperative hemoglobin in ANH group was higher than that in ABT group, and the difference was statistically significant (*P* < 0.05). There was no significant difference in preoperative coagulation function between the two groups, and postoperative APTT values were prolonged in the ANH group compared with the ABT group, and the difference was statistically significant (*P* < 0.05), as shown in Table 2.

### Platelet activating factor

The positive percentages of platelet and platelet activating factor expression measured at five time points from T0 to T4 in the central laboratory of our hospital showed that there was no significant difference in the expression of platelet and platelet activating factor before surgery between the two groups (P > 0.05). Platelet was significantly higher in the ANH group than in the ABT group from T2 to T4 (P < 0.05). At T1, the positive expression rate of platelet activating factor PAC-1 in ABT group was higher than that in A NH group (P < 0.05); the positive expression rate of platelet activating glycoprotein CD62P in ABT group was higher than that in AN H group at T3 and T4, and the difference was statistically significant (P < 0.05), as shown in Table 3.

**Table 3:** Comparison of platelet activating factor at each time point between the two groups

Group		ABT Group	ANH Group	t	*P*
PAC-1 (%)	T0	3.27 ± 0.11	3.28 ± 0.07	-0.175	0.861
	T1	3.10 ± 0.14	3.46 ± 0.12	- 10.804	<0.001 *
	T2	5.05 ± 0.19	5.26 ± 1.02	-0.813	0.420
	T3	5.10 ± 0.13	4.89 ± 1.04	1.189	0.239
	T4	4.78 ± 0.12	4.87 ± 0.70	-0.854	0.397
	T0	6.65 ± 0.14	6.63 ± 0.09	0.785	0.436
CD62P (%)	T1	6.54 ± 0.15	6.32 ± 0.89	1.371	0.176
	T2	7.03 ± 0.15	6.97 ± 1.27	0.254	0.800
	T3	7.18 ± 0.14	6.69 ± 1.20	2.208	0.031
	T4	6.84 ± 0.11	6.07 ± 0.85	4.850	<0.001
	T0	2 19.7 ± 3 8.5	2 08.2 ± 2 1.8	1.424	0.160
P lt (× 10 ^ 9/L)	T1	2 05.2 ± 2 2.5	1 95.9 ± 1 4.7	1.898	0.063
	T2	1 76.0 ± 2 3.3	2 01.2 ± 1 1.9	-5.261	<0.001
	T3	1 77.0 ± 2 2.3	1 97.0 ± 1 2.3	-4.301	<0.001
	T4	1 85.2 ± 3 3.2	2 21.1 ± 6 8.8	-2.574	0.013

Note: PAC-1 in ANH group was higher than ABT group at T1 *P* < 0.05; CD62P in ANH group was lower than ABT group at T4 *P* < 0.05; Plt in ANH group was higher than ABT group at T2 and T3 *P* < 0.05

### Lactic acid determination and hospital stay

There was no significant difference in preoperative lactic acid content between the two groups (*P* > 0.05). There was no significant difference in the hospital stays between the two groups (*P* > 0.05), as shown in Table 4.

**Table 4:** Comparison of lactic acid content and hospital stays between the two groups

Group	ABT Group	ANH Group	t	*P*
Preoperative lactic acid (mmol/L)	0.79 ± 0.26	0 .96 ± 0 .41	- 1.937	0.058
48 h lactate (mmol/L) after surgery	1.38 ± 0.67	1.34 ± 0.63	0.196	0.845
Length of stay (days)	5.7 ± 0.9	5.8 ± 1.0	- 0.538	0.593

### Adverse reactions and prognosis

The patients in the two groups had no obvious adverse reactions during and after operation, and were discharged from the hospital after postoperative rehabilitation.

## Discussion

In terms of THA for elderly patients, intraoperative bleeding, blood conservation, and changes in coagulation function are common concerns of orthopedics and anesthesiology, and they have close correlations with the prognosis of patients 4.

For many years, ABT and ANH have been used safely in operations with a large volume of blood loss. In the present research, the blood conservation techniques of ABT and ANH were performed on the selected 60 elderly THA patients with approximate bleeding volumes, respectively, during the operation, and the expressions of platelet activating factors before, during and after the operation, as well as changes in coagulation function of patients, were mainly observed, thereby exploring the strengths and weaknesses of different blood conservation techniques for patients.

### Hemoglobin content

The hemoglobin content in ANH group was higher than that in ABT group at 48 h after the operation. As revealed by the research of Quispe-Fernandez *et al.*, 5 supplementing the red blood cells lost during the operation can improve both the oxygen-carrying capacity and tissue oxygen supply. In ABT group, the surgical field blood was recovered with the automatic mode of the autologous blood recovery machine, washed, centrifuged, filtered, and then transfused back to the body of patients. In ANH group, autologous whole blood was transfused back during the operation. The two groups had no significant differences in the blood volume of transfusion. However, the content of effective red blood cells in the finished products of ABT group was not measured, which may be one of the reasons for the lower postoperative hemoglobin level compared with ANH group. The influence of the blood recovery machine on cell components was not involved in the present research, and further experiments were needed to find out the reasons.

### Coagulation function

Before the operation, the two groups exhibited no significant difference in coagulation function. At 48hours after the operation, the APTT value was larger in ABT group than that in ANH group, while neither of them exceeded the normal range. It indicated that in the experiment, the coagulation function of the 60 elderly patients subjected to the two blood conservation techniques during THA, respectively, could recover to the normal state at 48hours after the operation. APTT reflects the coagulation activity of the endogenous coagulation system. The autologous whole blood of ANH group was stored before the operation, which contained more coagulation substances compared with the washed and filtered autologous blood of ABT group 6, thus, better preserving the endogenous blood coagulation factors. This may lead to the decrease in the APTT value in ANH group.

### Determination of platelet activating factors

Platelet activating factors, which can act on many kinds of cells and tissues, have an in vivo biological activity similar to the hormone. As found by Lordan *et al.*^7^, Platelet activating factors play a mediating role in various cellular responses and have close associations with many cardiovascular diseases. Ramakrishnan et al. 8 also reported that intravenous injection of such factors in the body of rats can cause not only submucosal vasoconstriction, hemoconcentration, and reduction in the mucosal blood flow, but also the decrease in blood pressure, pulmonary hypertension, coronary artery constriction, cardiac function inhibition and metabolic acidosis. Moreover, platelet activating factors are also involved in gastric ulcer, ischemic gastric and small intestinal mucosal damage. Besides, they also play an important role in acute pancreatitis and its mediated lung injury, as well as system inflammatory reaction syndrome (SIRS) 9.

In the present research, it was found that ANH group displayed higher content of platelet than ABT group from T2 to T4, possibly caused by the transfusion of autologous whole blood that contained platelet in ANH group. Nonetheless, the two groups had similar results of PAC-1 expression, revealing that excessive platelet activation will not be caused after the operation by the transfusion of either washed and filtered AB or autologous whole blood. In addition, PAC-1 in ANH group was transiently higher than that in ABT group at T1, which may be related to the temporary activation of some platelets by the dilution of the collected autologous blood in ANH group 10.

## Conclusion

CD62P is a glycoprotein expression after platelets are activated, and its biological role has been highlighted by increasing researchers. Its pathophysiological effects have been demonstrated by extensive research, such as having close correlations with the hypercoagulable state of patients 11, acting as a major coordinator of inflammatory and immune responses 12, serving as one of the independent predictors of ischemic stroke 13, and laying a pathological basis for acute myocardial infarction 14.

There is a close correlation between the accumulation of CD62P in banked blood and the acute lung injury after blood transfusion 15.

In this research, the content of CD62P was higher in ABT group than that in ANH group at T3 to T4, indicating that compared with the intraoperative transfusion of autologous washed red blood cells, that of the autologous whole blood may be safer for elderly patients at 24 to 48 h after the operation.

## Data Availability

The datasets used and analysed during the current study are available from the corresponding author on reasonable request.
